# Dataset for Evaluating the Production of Phonotactically Legal and Illegal Pseudowords

**DOI:** 10.1038/s41597-025-05127-0

**Published:** 2025-05-14

**Authors:** Valérie Chanoine, Snežana Todorović, Bruno Nazarian, Jean-Michel Badier, Khoubeib Kanzari, Andrea Brovelli, Sonja A. Kotz, Elin Runnqvist

**Affiliations:** 1 Aix Marseille Univ, CNRS, LPL, Aix-en-Provence, France; 2 Aix-Marseille Univ, ILCB, Aix-en-Provence, France; 3 Institute of Psychology, Jagiellonian University, Kraków, Poland; 4 Institut de Neurosciences de la Timone UMR 7289, Aix Marseille Université, CNRS, 13005 Marseille, France; 5 Aix Marseille Univ, INSERM, INS, Marseille, France; 6 Department of Neuropsychology and Psychopharmacology, Maastricht University, Maastricht, The Netherlands; 7 Department of Neuropsychology, Max Planck Institute for Human Cognitive and Brain Sciences, Leipzig, Germany

**Keywords:** Language, Neurology

## Abstract

The “MEG-GLOUPS” dataset offers a curated collection of raw magnetoencephalography recordings from seventeen French participants engaged in a pseudoword learning task as well as resting-state activity before and after the task. A dataset called Gloups with the same participants and a similar learning task adapted to functional magnetic resonance imaging is already available. In the learning task, participants were instructed to pronounce monosyllabic pseudowords, which were presented both visually and auditorily. These pseudowords were either phonotactically legal or illegal in the participants’ native language, French. We organized the dataset according to the Brain Imaging Data Structure (BIDS), pre-processed the data and performed a minimal analysis of Event-Related Fields (ERFs), to ensure data quality and integrity of the dataset. This data collection includes comprehensive descriptions of the theoretical background, methods, data recordings, and technical validation.

## Background & Summary

Understanding the neural mechanisms sustaining the production and learning of speech motor sequences is important for models of speech and language production, as well as for understanding the adaptive error monitoring in speech and language acquisition. Several studies have explored this topic using fMRI^1–4^ where participants learned to pronounce pseudowords (word-like strings of sounds without meaning) made up of syllables that either are possible (phonotactically legal) or not (phonotactically illegal) in their native language. Learning to produce phonotactically illegal pseudowords with novel syllables needs close monitoring, but such learning needs to be controlled by also looking at a condition where learning and monitoring is not required. Thus, by contrasting phonotactically illegal and legal pseudoword production, processes involved in production, monitoring, and learning of speech motor sequences can be isolated. However, a precise explanation of the dynamics related to speech production at this post-lexical stage as well as of the adaptive processes facilitating learning is still missing. In the current study we wanted to highlight the spatio-temporal dynamics associated with the learning and production of new speech motor sequences. To this end, we conducted a magnetoencephalography (MEG) experiment, generating the “MEG-GLOUPS” dataset that provides fine grained temporal resolution in an attempt to differentiate these different processes. Participants produced pseudowords with a phonotactic structure that either followed the rules of their native language or not (i.e., phontactically legal or illegal). These pseudowords were repeated multiple times over the course of the experiment. This approach to speech motor sequence learning can not only showcase learning over time at specific stages of speech production but also informs other research that explores monitoring and adaptive control.

Several previous studies on monitoring and adaptive control focused on the processing of auditory feedback generated during speech production. Numminen *et al*.^5^ reported that reading aloud as opposed to listening to one’s own speech resulted in an attenuated response of an evoked component around 100 ms after stimulus onset (M100). This finding was related to participants’ ability of predicting the sensory consequences of self-produced speech (i.e., efference copy), which cancels out an anticipated sensory response (i.e., reafference cancellation). Modulations of reafference cancelation in response to mismatches between predicted sensory and actual feedback provide speakers with an error signal used to monitor and adaptively control successful speech production. Several studies have replicated this phenomenon using very simple speech targets such as single vowels. M100 amplitude modulations in response to diverse manipulations of sensory feedback related to self-produced speech (addition of noise or tones in the feedback^6^; pitch-shifted and alien speech feedback^7^; parametric delays or pitch shifted feedback to covert articulation^8^) are consistent with the idea that the M100 modulation reflects the comparison of predicted and actual sensory feedback. Ventura *et al*.^9^ observed that the rate and complexity of the speech target also modulates the M100 amplitude: when speech rate and complexity increase, speech production might become less predictable and the difference between speaking and listening reduces the M100 amplitude. Niziolek *et al*.^10^ reported less reafference cancellation for atypically compared to typically produced vowels, which suggests that the efference copy might reflect higher-level (e.g., phonemic) properties of target sounds and not simply the specific motor commands used to generate them. The current study, investigating the production of multiphonemic speech targets differing in complexity that over the course of the experiment presumably became less variable and more predictable, could thus provide further information about the dynamics of reafference cancellation-based adaptive control.

Concerning the time course of speech production, several studies have used MEG focusing on planning processes when producing words and phrases. Concerning post-lexical processes in a picture naming task, Strijkers *et al*.^11^ observed activity in the motor and the posterior superior temporal cortex reflecting articulatory-acoustic phonological features ( + LABIAL vs. + CORONAL) of word-initial speech sounds (e.g., Monkey vs. Donkey). This articulatory-acoustic effect was significant in an early time window (160–240 ms post stimulus onset), temporally coinciding with a fronto-temporal lexical frequency effect. Carota *et al*.^12^ also used a picture naming task but observed a progression of neural activity from anterior to posterior language regions for semantic and phonological/phonetic computations preceding overt speech. Stimulus-locked spatiotemporal responses to object categories started around 150 to 250 ms after picture onset, whereas word length was decoded at left frontotemporal sensors around 250–350 ms followed by phonological neighborhood density (350–450 ms). The focus on syllabification in the current study complements the previously described studies by informing on the time course associated to a different post lexical processes. Moreover, as syllabification is a combinatorial process, it might also complement previous literature on higher-level combinatorial processes. For instance, Pylkkänen *et al*.^13^ reported effects of combinatorial processing in the ventro-medial prefrontal cortex (vmPFC) and left anterior temporal lobe (LATL) for phrase production (i.e., combining and adjective and a noun such as “the red car”). These effects showed relatively early (180 ms) after the presentation of a production prompt, suggesting that combination commences with initial lexical access. The current task, which was devoid of lexical and semantic information and isolated a post-lexical process, might therefore shed light on what is unique or common to combinatorial processes at different linguistic levels.

The “MEG-GLOUPS” dataset offers a curated collection of raw magnetoencephalography recordings from seventeen French participants engaged in a syllable learning task involving the overt production of phonotactically legal and illegal pseudowords. We also collected resting state data before and after the task. A functional magnetic resonance imaging dataset called Gloups (same participants) is already available (OpenNeuro ds004597).

This data descriptor describes the contents of the dataset. We time-stamped the onset of each pseudoword in the metadata of the recordings. Each pseudoword was labelled with a trial type based on its phonotactic legality (legal versus illegal), sequence order (one to nine), and participant response accuracy (correct vs. incorrect).

We structured the dataset according to the Brain Imaging Data Structure (BIDS) standard and proposed a preliminary Event-Related Fields (ERFs) analysis. Prior to this, we applied appropriate methods, including rigorous data cleaning procedures, to mitigate artifacts caused by environmental and physiological noise, further ensuring the quality and integrity of the dataset

This data collection includes comprehensive descriptions of the theoretical background, methods, data recordings, and technical validation.

## Methods

### Participants

Seventeen native French speaking adults are available for further data analysis (11 females with age: M = 25.7, SD = 3.5 and 6 males with age: M = 26.3, SD = 3.9). Participants were screened by a medical doctor for medical contraindications and reported having normal hearing and no history of neurological disorders. In addition, screening prior to the experiment ensured that no participant had knowledge of a language that included syllable types used in the phonotactically illegal experimental condition. The experiment was conducted in accordance with the Declaration of Helsinki and with the understanding and written consent of all participants. The study received ethical approval (filed under Id 2017-A03614-49 from the regional ethical committee, Comité de protection des personnes sud Méditerranée I).

### Procedure

The MEG data acquisition began and finished with a 3-minute resting state recording. During this period, participants were instructed to keep their eyes open, avoid thinking about anything specific, and minimize movement. Between these two resting state runs, the experiment included nine 6-minute-long learning task sequences, evenly distributed across three runs. Each pseudoword was repeated 45 times (5 repetitions x 9 sequences).

**Fig. 1:**
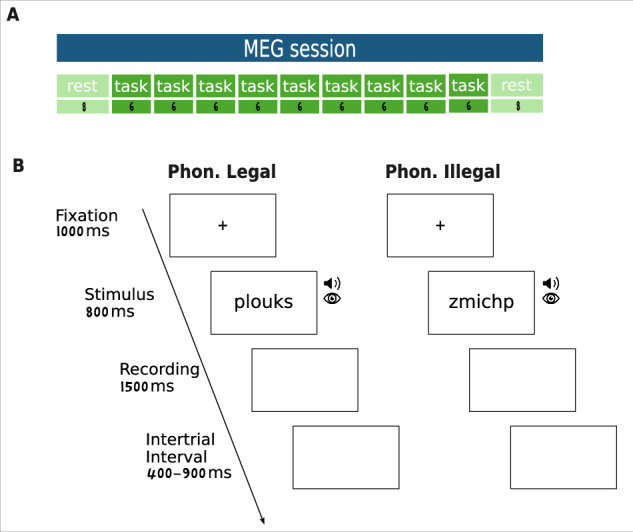
Experimental Design. (**A**) The MEG session begins and ends with a resting-state task lasting 8 minutes. Between the two resting-state periods, 9 blocks of experimental tasks (learning task) are presented. (**B**). Depiction of an experimental trial in the learning task. The learning task involved pronouncing novel pseudowords composed of phonotactically legal or illegal syllables, presented in both visual and auditory modalities.

For the learning task, pseudowords remained on the screen for 800 ms and were synchronously presented visually and auditorily for 660 ms. They were preceded by a fixation cross for 1000 ms and followed by a blank screen during which participants were instructed to pronounce the presented pseudoword. The time allotted for pronunciation was 1500 ms. The jittered inter-stimulus interval ranged from 400 to 900 ms. On average, a single trial lasted 4 seconds. Figure 1 shows an example trial. The order of presentation was pseudo-randomized. Eight randomization lists were created to prevent order effects both within and across subjects and across MRI and MEG sessions (see /sourcedata/stimuli in Data Records section).

Stimulus presentation was controlled by a custom-made software compiled using the LabVIEW 2020 development environment (National Instruments). The audio layer was composed by an opticalp microphone (Sennheiser MO2000CU) and a pneumatic sound delivering system driven by a Stax electrostatic amplifier using earplugs. The software enabled pre-experiment testing of the sound system, headphones, and microphone, as well as the recording of vocal productions during the experiment. Trials were recorded using a multifunction NI PCIe-6353 DAQ (National Instruments) and stored as individually labeled WAV files (22050 Hz, 16-bit mono). Audio stimuli were delivered via a NI-6212 multifunction board (National Instruments), leveraging its high-precision analog output—preferred over the laptop’s built-in audio card for reliability. The stimuli, broadcast at 22050 Hz, were triggered via the NI-6212’s digital port, which also sent labeled triggers to the MEG imager in parallel. A benchmark test confirmed millisecond-accurate audio onset*. Participants’ vocal responses were recorded with the same precision using the Sennheiser MO2000CU microphone, connected to a differential analog input on the NI-6212 board*.

### Stimuli

The stimuli were the same as those described in Todorović *et al*.^4^. Target stimuli were 36 CCVCC syllables (C – consonant, V – vowel) composed of French phonemes. In one half of the stimuli, these phonemes were combined in a phontactically legal way in French. The other half of the stimuli were syllables consisting of novel combinations of phonemes (phonotactically illegal^1^. Two-consonant clusters were selected from the French language database lexique.org^14^ and filtered for their frequency of occurrence at the beginning or at the end of the syllable^15^. The clusters with high frequency (141,6 ± 251,6) were used to form phonotactically legal stimuli, and those with a frequency close to zero (0,3 ± 0,8) to form phonotactically illegal stimuli. The resulting syllables were checked for orthographic neighbors using WordGen software^16^. Only one stimulus had one orthographic neighbor (spald), the rest of stimuli had none.

All stimuli were recorded by a Serbian speaker, as Serbian allows pronunciation of all used combinations. Stimuli were also visually presented according to French orthographic rules. As this experiment was part of a larger study that took place over two sessions (one fMRI and one MEG session), any given participant was only presented with half of the stimuli in a counterbalanced manner.

### Anatomical MRI data acquisition

The experiment was conducted on a Siemens Magneton Prisma 3 T scanner at the Centre IRM-INT@CERIMED (UMR 7289 CNRS–Aix-Marseille University) using a 64-channel head coil. Whole-brain anatomical magnetic resonance imaging (MRI) data were acquired using high-resolution structural T1-weighted image (MPRAGE sequence, voxel size = 1 × 1 × 1 mm^3^, data matrix 256 × 256, TR/TI (inversion time)/TE = 2,300/900/2.98 ms, flip angle = 9°).

The anatomical images are available on OpenNeuro Dataset ds004597 (Gloups, https://openneuro.org/datasets/ds004597/versions/2.0.0) and are linked to the reference article^4^. They were organized according to the Brain Imaging Data Structure^17^ (BIDS) and MNE-BIDS framework^17,18^. To prevent errors due to inconsistencies in participant identifiers across techniques, the list of MRI and MEG identifiers for each participant is provided in Table 1.

**Table 1:** Participants’ identifiers.

Participants			
ID-MEG	Gender	Age	ID-IRM
sub-01	F	24	sub-04
sub-03	F	27	sub-08
sub-04	F	24	sub-13
sub-05	F	28	sub-15
sub-06	F	22	sub-14
sub-07	M	26	sub-09
sub-08	F	27	sub-06
sub-10	F	27	sub-19
sub-12	M	34	sub-17
sub-13	F	21	sub-12
sub-14	M	23	sub-21
sub-15	M	25	sub-03
sub-16	F	28	sub-25
sub-17	F	33	sub-24
sub-18	M	24	sub-01
sub-19	F	22	sub-23
sub-20	M	26	sub-22

Correspondence between MEG (ID-MEG) and MRI (ID-IRM) identifiers. The participants’ gender and age are also indicated.

### MEG data acquisition

The experiment was conducted using a 4D Neuroimaging Magnetometers 248-channel scanner (Timone Hospital, Marseille, France). MEG data were recorded continuously with a sampling rate of 2034.51 Hz.

Head shape and position coil location were recorded using a Polhemus Fastrak 3-D digitizing stylus at the beginning of the recording run (5 runs per participant, i.e., two resting state runs, and three learning task runs). We ensured that the position of the sensor regarding the subject did not change during the run and between the runs more than 3 mm. The head shape obtained from the digitization of the head allows checking and eventually compensate for differences in head position between runs or to match to the participant’s MRI.

Electrooculogram (EOG) and electrocardiogram (ECG) were recorded simultaneously (using a 256-channel BrainAmp amplifier system, Brain Products) with a sampling rate of 2500 Hz for the offline rejection of eye movements and cardiac artefacts.

All stimuli were presented to participants on a mirror by a back-projection system where an LCD projector was placed outside the magnetically shielded room to avoid interfering electrical apparatus.

The distance between the participant’s eyes and the screen on which stimuli were displayed was similar across participants.

A trigger square invisible to the participant was projected onto a photodiode which was used to signal the presence of a stimulus on-screen and to synchronize the MEG and EOG/ECG recordings.

### Behavioural Data Processing and Analyses

**Fig. 2:**
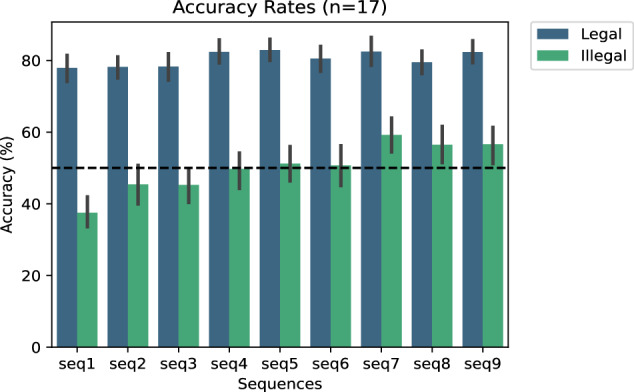
Averaged accuracy rates across all subjects (n = 17). Accuracy rates are presented as percentages, plotted against two factors: phonotactic legality (Legal in blue vs. Illegal in green) and sequence order (ranging from 1 to 9). The black dashed horizontal line indicates the chance level at 50%.

All pseudoword productions were labelled as incorrect if they contained insertions, omissions, hesitations, or self-repairs, if they were impartial or missing, or if a pseudoword was pronounced as two or more syllables. They were labelled as correct in all other cases. A preliminary analysis was conducted based on individual accuracy scores (0 for incorrect and 1 for correct responses) for each trial and sequence. Figure 2 provides an overall view of the averaged accuracy rates across all participants (n = 17), considering two experimental factors: phonotactic legality (legal vs. illegal) and stimulus presentation order (Sequence order).

Accuracy scores varied significantly depending on the phonotactic legality of the stimuli. For phonotactically legal pseudowords, participants performed well above 70%, both in the initial and final learning sessions. In contrast, for phonotactically illegal pseudowords, performance barely exceeded chance level (50%) during the first three learning sequences but then substantially surpassed this threshold in the next sequences. At the individual level, only one participant (sub-12) exhibited markedly different behaviour, performing at chance level regardless of the phonotactic legality of the pseudowords (see Table 2).

**Table 2:** Behavioural Results.

	Accuracy Rate	
Subject	Illegal	Legal
sub-01	72.1	87.4
sub-03	56.0	87.7
sub-04	55.6	92.4
sub-05	52.6	81.0
sub-06	60.0	88.4
sub-07	69.4	73.1
sub-08	43.2	79.5
sub-10	8.6	67.7
sub-12	17.5	**45.1**
sub-13	52.3	88.9
sub-14	81.5	83.7
sub-15	38.5	71.4
sub-16	53.6	89.1
sub-17	44.0	84.0
sub-18	46.7	85.2
sub-19	64.7	83.7
sub-20	42.7	86.2

Accuracy rates (in percent) per participant and per Legality factor (phonotactically legal versus illegal stimuli). Only Subject 12 exhibits scores below the chance level for Legal stimuli.

### MEG data Preparation

For the learning task, the trigger values in the raw MEG files were modified based on the participants’ response accuracy (correct or incorrect responses for each stimulus). Initially, the trigger values were used to distinguish the stimuli based on their phonotactic legality (legal or illegal) and their presentation order (per block, ranging from 1 to 5, and per sequence, ranging from 1 to 9). To account for response accuracy, the trigger values in the raw MEG files were modified. Table 3 provides a detailed enumeration of the triggers after modification. To harmonize the MEG files, the EOG electrodes recording vertical and horizontal movements were renamed to EEG061 and EEG062, respectively. In the end, the raw 4D MEG files underwent the transformation of the trigger values and the EOG names before being converted to the FIF and BIDS formats.

**Table 3:** Trigger Values.

Label	Value	Label	Value
Legal_Correct_1	524	Illegal_Correct_1	526
Legal_Correct_2	534	Illegal_Correct_2	536
Legal_Correct_3	544	Illegal_Correct_3	546
Legal_Correct_4	554	Illegal_Correct_4	556
Legal_Correct_5	564	Illegal_Correct_5	566
Legal_Correct_6	574	Illegal_Correct_6	576
Legal_Correct_7	584	Illegal_Correct_7	586
Legal_Correct_8	594	Illegal_Correct_8	596
Legal_Correct_9	604	Illegal_Correct_9	606
**Label**	**Value**	**Label**	**Value**
Legal_Incorrect_1	528	Illegal_Incorrect_1	530
Legal_Incorrect_2	538	Illegal_Incorrect_2	540
Legal_Incorrect_3	548	Illegal_Incorrect_3	550
Legal_Incorrect_4	558	Illegal_Incorrect_4	560
Legal_Incorrect_5	568	Illegal_Incorrect_5	570
Legal_Incorrect_6	578	Illegal_Incorrect_6	580
Legal_Incorrect_7	588	Illegal_Incorrect_7	590
Legal_Incorrect_8	598	Illegal_Incorrect_8	600
Legal_Incorrect_9	608	Illegal_Incorrect_9	610

The table outlines the modified trigger values (common base value = 512), adjusted according to the factors of Legality/Participant Accuracy (+2 = Legal correct, +4 = Illegal correct, +6 = Legal incorrect, +8 = Illegal incorrect) and sequence number (+10 for Sequence 1, up to + 90 for Sequence 9).

For the resting-state data, no modifications were made to the trigger values before converting the raw 4D MEG files to the FIF and BIDS formats, only the EOG electrode names were changed.

Table 4 presents the distribution of runs for each participant based on the MEG (Learning task and Resting-state). For two participants, one run is missing (Run 2, i.e., the first learning task run for participant 10 and Run 5, i.e., the second resting state for participant 17). Three participants have their learning task distributed across four runs instead of three (participants 7, 16 and 19).

**Table 4:** Runs.

Subject	RUNS	
	Learning	Resting-state
sub-01	2,3,4	1,5
sub-03	2,3,5	1,6
sub-04	3,4	1,5
sub-05	2,3,4	1,5
sub-06	2,3,4	1,5
sub-07	2,3,4,5	1,6
sub-08	2,3,4	1,5
sub-10	*,3,4	1,5
sub-12	2,3,4	1,5
sub-13	2,3,4	1,5
sub-14	2,3,4	1,5
sub-15	7,8,9	6,10
sub-16	3,4,5,6	2,7
sub-17	4,5,6	2,*
sub-18	2,3,4	1,5
sub-19	2,3,4,5	1,6
sub-20	2,3,4	1,5

Distribution of runs for each participant in the Learning and Resting State tasks.

## Data Records

The dataset is organized according to MNE Brain Imaging Data Structure (MNE BIDS^5,6^) version 1.7.0 and publicly available on OpenNeuro (accession number ds005261)^19^ under a Creative Commons Licence 0.

**Fig. 3:**
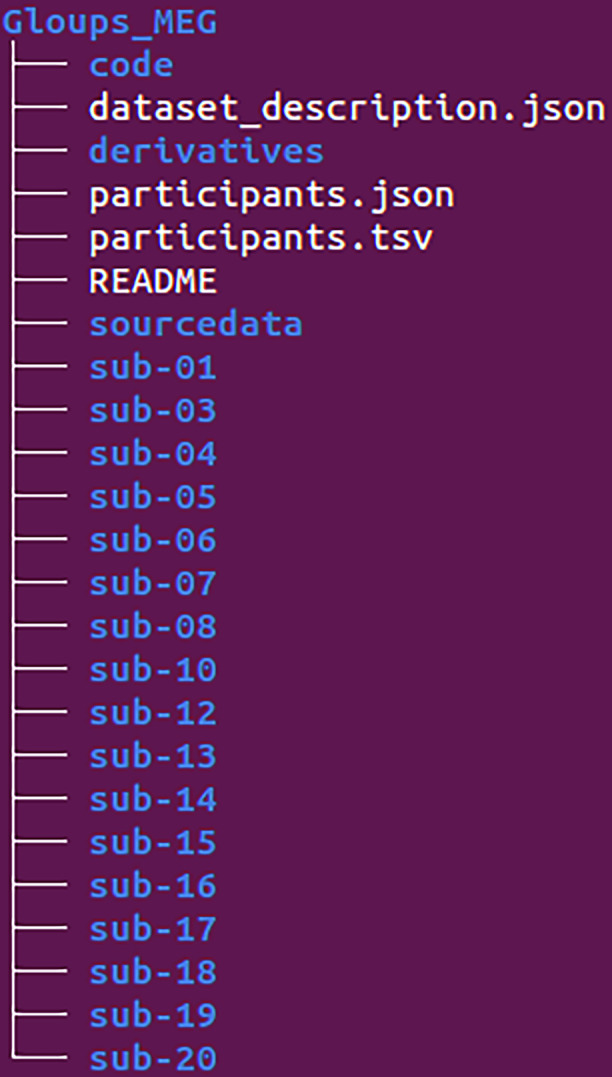
BIDS Dataset Tree. Global representation of the BIDS dataset directory structure (the dataset root is ‘Gloups_MEG’).

In this section, we provide a more detailed description of the dataset structure and its contents. The /Gloups_MEG directory (see Fig. 3) serves as the root directory of the dataset. It contains 17 participant directories with raw data, each labeled with the prefix sub- followed by a two-digit identifier. Additionally, it includes the /code, /derivatives, and /sourcedata directories, along with the mandatory BIDS files, such as the README, participants list, and dataset description.

**Fig. 4:**
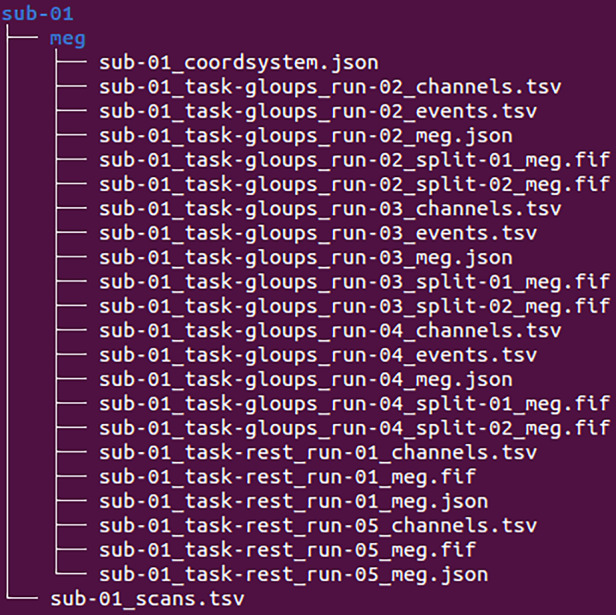
BIDS Tree Subset. Truncated representation of the BIDS directory structure for one participant directory (Subject 01).

The participant folders contain the MEG directory. An example with one participant (sub-01) is shown in Fig. 4. This directory hosts the raw MEG files for the Learning task (task-gloups) and the Resting-State task (task-rest), organized by runs. It also includes files related to the description of event markers (_events.tsv), the order and properties of the channels (_channels.tsv), and the coordinate system used for the MEG, EEG, head localization coils, and anatomical landmarks (_coordsystem.json).

However, the raw MEG files for the Learning task have undergone minimal preprocessing to facilitate the handling of correct and incorrect trials, as described in the MEG data Preparation section.

**Fig. 5:**
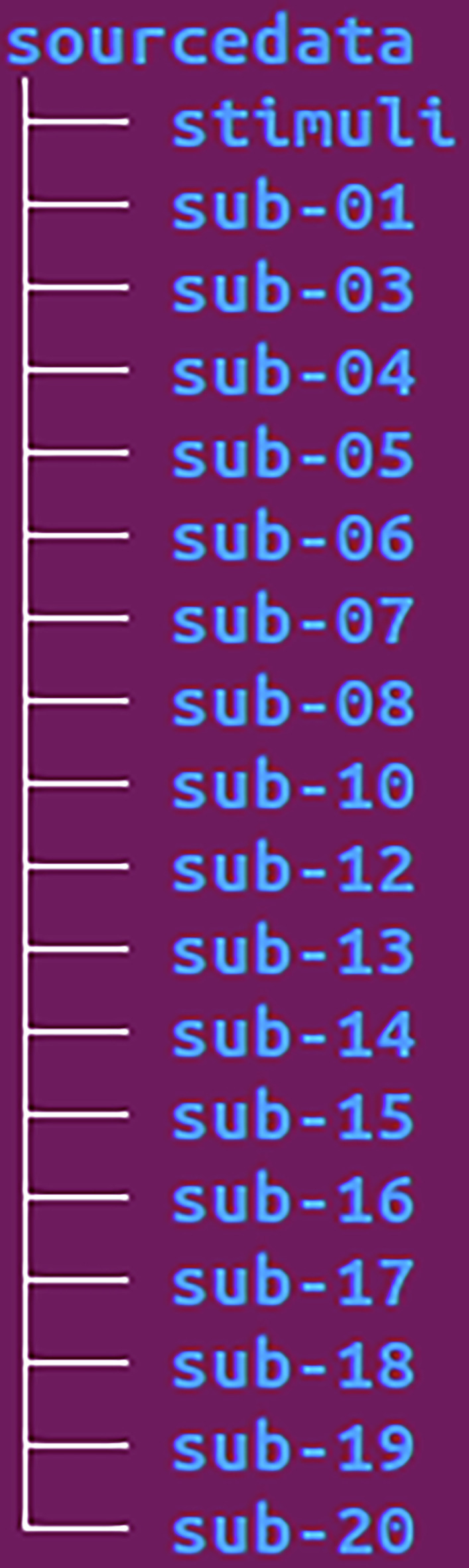
BIDS Tree Sourcedata. Representation of the BIDS directory structure for the /sourcedata directory.

The /sourcedata directory (Fig. 5) contains both the /stimuli folder and the participants’ directories.

The /stimuli folder includes the WAV files used as stimuli for the Learning task, as well as DESC files that document the stimulation parameters, following the format recommended by the LabVIEW software. The filenames of the DESC files specify the name of the task, the sequence number (ranging from 1 to 9) as well as the randomization list, chosen from eight possible options: A1, A2, B1, B2, C1, C2, D1, D2 (e.g., task-gloups_seq 1_rand-A1_desc)

The participant’s /sourcedata directory includes the log files from the LabVIEW software with the specific parameters of stimulation and responses to each stimulaition (WAV files).

**Fig. 6:**
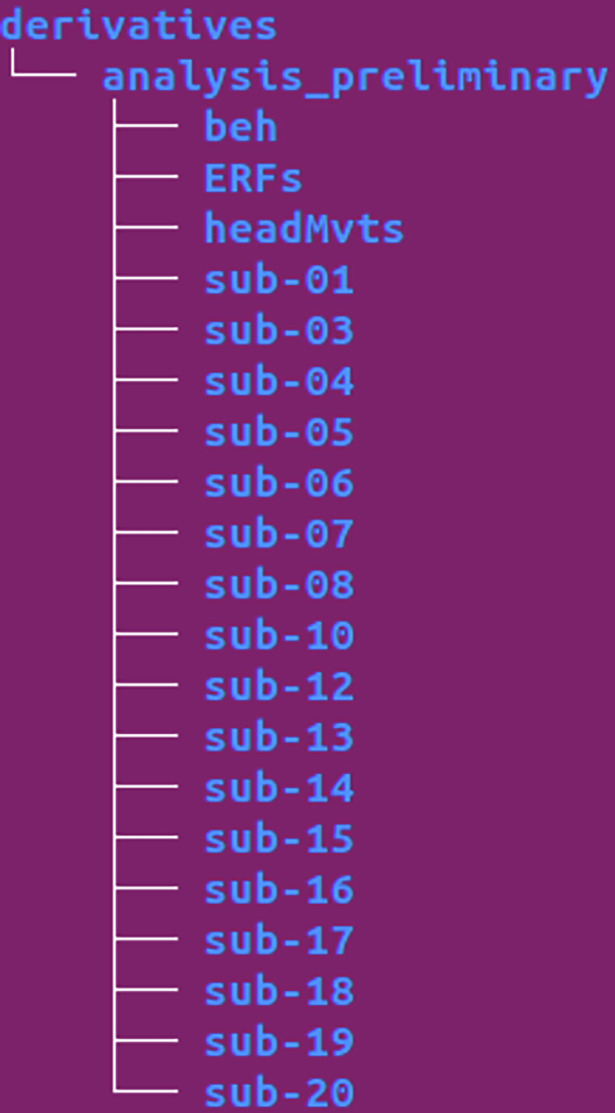
BIDS Tree Derivatives. Representation of the BIDS directory structure for the /derivatives directory.

The /derivatives directory (Fig. 6) contains all files derived from our preliminary analysis and is organized into a folder called analysis_preliminary. This directory includes the /beh,/ERFs, and /headMvts folders, as well as participant-specific directories.

In the /analysis_preliminary/beh directory, there is a summary table of accuracy results for all participants. The success rates are categorized by subject, sequence, and phonotactic legality. This table is stored as a TSV file and was generated using the script /code/03_MEG_GLOUPS_Accuracy_Rates.py.

In the /analysis_preliminary/headMvts directory, there is a TSV file that records head movement displacement in millimeters. This file was generated using the script code/05_MEG_GLOUPS_ComputeHeadMovements.py.

In the /analysis_preliminary/ERFs directory, the figures (in SVG format) related to the ERF analyses performed on all subjects are stored. All these figures were generated by the script /code/10_MEG_GLOUPS_GrandAveraging.py.

The participant’s /analysis_preliminary/meg directory contains the preprocessed MEG data and their sidecar files, in accordance with the results of all technical validation pipelines and Event-Related Fields (ERFs) analysis.

**Fig. 7:**
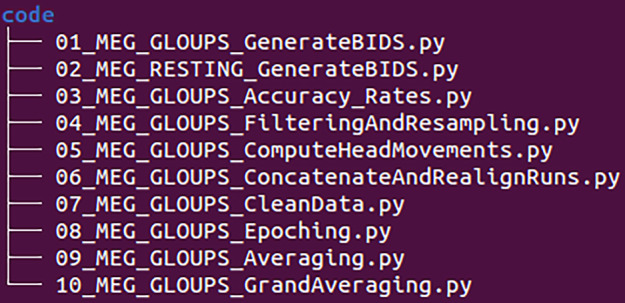
BIDS Tree Code. Representation of the BIDS directory structure for the /code directory.

Finally, as shown in Fig. 7, the /code directory contains the Python scripts used for the technical validation.

## Technical Validation

### We checked that the present dataset complies with the standardized brain imaging data structure by using the Python Bids-Validator library (version 1.15)

The processing of the present data is based on the free and open-source ecosystem of the neuroimaging community. We mainly used:

- MNE BIDS (https://mne.tools/mne-bids)
- MNE-python (https://mne.tools/stable/index.html)

### Behavioral data

Raw behavioral data (log files from Labview) were collected in the participant’s /sourcedata directory.

These data consist of TXT files that adhere to the following naming convention: beh_{sub}_{task}-{random}-[seq}_{run]_{date}.txt, where sub refers to the the participant’s identifier, task refers to the task name, random is the randomization code, seq is the sequence number (ranging from 1 to 9), and run indicates the run number (from 1 to 9, applicable only to the behavioral files, and distinct from the run numbering used for MEG files). To avoid any confusion, the term “sequence” will be used in behavioral data analyses to refer to the order of stimulus presentation, while the term “run” will be exclusively used for MEG data analyses.

The behavioral data were pre-processed in Python for the calculation of accuracy scores. The process involved the following steps:

1. Collection of Accuracy Scores: Participants’ scores (1 for correct responses and 0 for incorrect responses were added as the last column in the corresponding behavioral file located in the participant’s /sourcedata folder, preserving the order of stimulus presentation. The resultant behavioral files were then copied to the participant’s /analysis_preliminary/beh folder in accordance with BIDS recommendations.
2. Calculation of Accuracy Rates: The results of the behavioral analyses (accuracy rates) per participant, per phonotactic legality of the stimuli (legal or illegal) and per sequence order were stored in a TSV file (Accuracy_perSubject_perSequence_perLegality.tsv) located in the /analysis_preliminary/beh directory.

The code used for calculating accuracy scores, generating summary tables, and figures is provided for reference in /code/03_MEG_GLOUPS_AccuracyRates.py.

### MEG Data Preparation

The MEG functional raw data were processed using MNE-BIDS library to align with the BIDS format and are stored in each participant’s /meg directory. For the learning task, the data preparation involved modifying the trigger values to reflect the following factors:

- Subject response accuracy (correct or incorrect),
- Phonotactic legality of the stimuli (legal or illegal),
- Presentation order of the stimuli (sequence ranging from 1 to 9).

To comply with BIDS guidelines, an Events.tsv file was added to each participant’s /meg directory. This file includes the required columns (onset, duration, and trial_type), with the trial_type column encoding the aforementioned factors to provide detailed contextual information for each event. The code used for BIDS conversion and event file creation was separated for each task: 01_MEG_GLOUPS_GenerateBIDS.py for the learning task and 02_MEG_RESTING_GenerateBIDS.py for the resting-state task.

### MEG Data Pre-processing

The MEG data were subsequently pre-processed to assess signal quality and provide a preliminary analysis in the form of Event-Related Fields (ERF). The processed data are stored in the participant’s /derivatives/analysis_preliminary/meg folder.

The pre-processing steps include the following:

#### Filtering and Resampling

- Signal Filtering: Applying 0.5–30 Hz band-pass filters to remove low-frequency drifts and high-frequency noise.
- Resampling: Downsampling the data to a sampling frequency of 250 Hz to reduce data size and computational load while preserving the relevant signal components.
  - Code: /code/04_MEG_GLOUPS_FilteringAndResampling.py
  - Input files: MEG FIF files in the participant’s /meg directory
  - Output files: MEG FIF files in the participant’s /derivatives/analysis_preliminary/meg directory (e.g. sub-01_task-gloups_run-02_Filt_0p5_30_sFreq_250-meg.fif)

#### Estimation of Head movements

The device-to-head transformation describes the head’s position relative to the MEG measurement system. This matrix was used to analyze changes in individual head position, particularly between two successive runs of the Learning task. The Euclidean distance was computed to quantify head displacements between consecutive runs. The resulting table (HeadDisplacements.tsv) provides head displacement values for each participant across runs (see Table 5).

- Code: /code/05_MEG_GLOUPS_ComputeHeadMovements.py
- Input files: filtered and resampled MEG FIF files in the participant’s /derivatives/analysis_preliminary/meg directory (e.g., sub-01_task-gloups_run-02_Filt_0p5_30_sFreq_250-meg.fif)
- Output files: TSV file in the /derivatives/analysis_preliminary/headMvts directory (HeadDisplacements.tsv)

**Table 5:** Head Movements.

Subject	Run1	Run2	Displacement
sub-01	2	3	0.8
sub-01	3	4	1.8
sub-03	2	3	1.1
sub-03	3	5	1.0
sub-04	3	4	0.3
sub-05	2	3	0.7
sub-05	3	4	1.2
sub-06	2	3	0.5
sub-06	3	4	1.6
sub-07	2	3	1.4
sub-07	3	4	1.4
sub-07	4	5	0.0
sub-08	2	3	0.8
sub-08	3	4	0.4
sub-10	3	4	0.4
sub-12	2	3	0.9
sub-12	3	4	0.5
sub-13	2	3	2.0
sub-13	3	4	0.8
sub-14	2	3	1.3
sub-14	3	4	2.8
sub-15	7	8	0.9
sub-15	8	9	1.3
sub-16	3	4	2.1
sub-16	4	5	0.2
sub-16	5	6	0.6
sub-17	4	5	**9.0**
sub-17	5	6	0.0
sub-18	2	3	0.6
sub-18	3	4	1.0
sub-19	2	3	**11.0**
sub-19	3	4	0.2
sub-19	4	5	0.0
sub-20	2	3	1.1
sub-20	3	4	0.9

Translation displacement (in millimetres) between two successive runs (Run1 and Run2) for each participant. A potential exclusion criterion for a given run in our study can be a displacement greater than 3 millimetres.

Table 5 reports excessive head movements (translation displacement greater than 3 millimeters) only between MEG runs 4 and 5 for subject 17 and between runs 2 and 3 for subject 19.

In the preliminary analyses, no runs were excluded, as a spatial realignment of MEG runs was performed to correct for this issue (see the following section).

#### Concatenation and Alignment of runs per Participant

Before concatenating MEG runs, we realigned them to a common head position using the Maxwell filtering algorithms with a single reference MEG run per participant. These algorithms have been adapted in MNE-Python from previous work^20,21^.

- Code: /code/06_MEG_GLOUPS_ConcatenateAndRealignRuns.py
- Input files: filtered and resampled MEG FIF files in the participant’s /derivatives/analysis_preliminary/meg directory (e.g., sub-01_task-gloups_run-02_Filt_0p5_30_sFreq_250-meg.fif)
- Output files: MEG FIF file in the participant’s derivatives/meg/ directory (e.g., sub-01_task-gloups_Filt_0p5_30_sFreq_250_AlignedRuns-meg.fif)

#### Cleaning the MEG continuous data

Cleaning the continuous MEG data involves homogenizing the names of the EOG and ECG channels, detecting and removing bad sensors using graphical assistance (interactive figure based on a power spectrum density calculation), and the removing physiological noise from eye movements or heartbeats using the Signal Space Projection technique^22^.

- Code: /code/07_MEG_GLOUPS_CleanData.py
- Input files: realigned MEG FIF files in the participant’s /derivatives/analysis_preliminary/meg directory (e.g., sub-01_task-gloups_Filt_0p5_30_sFreq_250_AlignedRuns-meg.fif)
- Output files: cleaned MEG FIF file in the participant’s /derivatives/analysis_preliminary/meg directory (e.g., sub-01_task-gloups_cleaned-raw.fif)

## Preliminary Analysis

### Data Segmentation or Epoching

Epoching was performed around the events of interest, based on triggers recorded simultaneously with the MEG data. Just before epoching, and to ensure that event signals were properly transmitted to the stimulation channels, a figure illustrating the distribution of all events in the continuous data was saved for each participant (e.g., sub-05_task-gloups_events.svg).

**Fig. 8:**
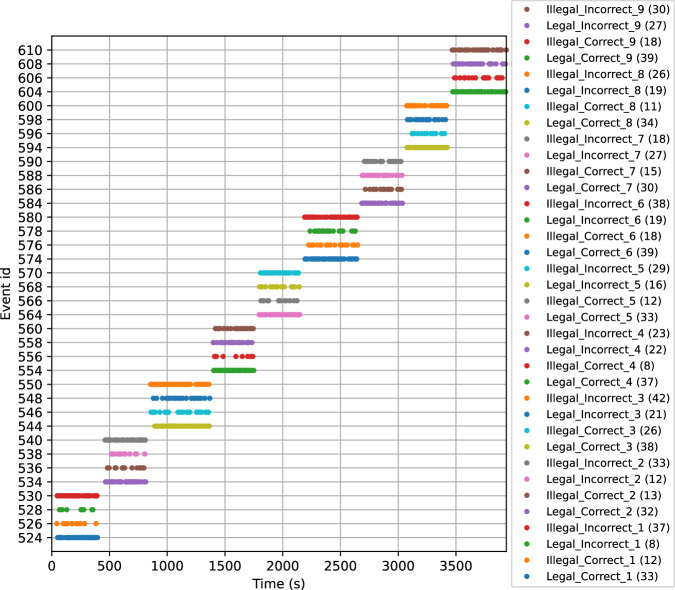
Events. Illustration of the event distribution as a function of trigger values.

**Fig. 9:**
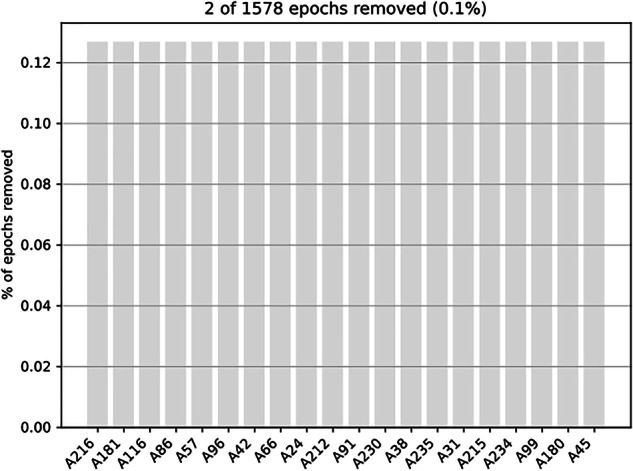
Rejected Trials. Drop Log Representation to evaluate the number of rejected trials across all epochs for a given participant.

Epochs were extracted within a time window of [−0.2 s to +2 s] relative to stimulus onset. Baseline correction was applied by subtracting the average pre-stimulus activity ([−0.2 s to 0 s]) from each epoch. Epochs identified as outliers based on signal fluctuations (rejection threshold: <−3000 fT or >3000 fT, flatness criterion: <1 fT or >−1 fT) were excluded. To assess the number of rejected trials, a “drop log” figure (e.g., sub-01_task-gloups_drop_log.svg) was saved for each participant. The _events.svg and _drop_log.svg figures are stored in each participant’s /derivatives/analysis_preliminary/figures directory (see respectively, Figs. 8 and 9).

- Code: /code/08_MEG_GLOUPS_Epoching.py
- Input files: cleaned MEG FIF files in the participant’s /derivatives/analysis_preliminary/meg directory (e.g., sub-01_task-gloups_cleaned-raw.fif)
- Output files: Epoched MEG FIF file in the participant’s /derivatives/analysis_preliminary/meg directory (e.g., sub-01_task-gloups-epo.fif) and SVG files for illustration of events and rejected trials in the participant’s /derivatives/analysis_preliminary/figures directory.

### Averaging and Event-related fields (ERFs)

For each participant, epochs were averaged across trials to extract the ERF signals for each condition of interest (Legal Correct, Legal Incorrect, Illegal Correct versus Illegal Incorrect). These steps ensure the reliability of the signal for further analysis and provide a first glance at the data quality through ERF visualization.

- Code: /code/09_MEG_GLOUPS_Averaging.py
- Input files: Epoched MEG FIF file in the participant’s /derivatives/analysis_preliminary/meg directory (e.g., sub-01_task-gloups-epo.fif)
- Output files: Averaged MEG FIF file in the participant’s /derivatives/analysis_preliminary/meg directory (e.g., sub-01_task-gloups-ave.fif) and corresponding SVG figures in the participant’s /derivatives/analysis_preliminary/figures directory

### Grand averaging

**Fig. 10:**
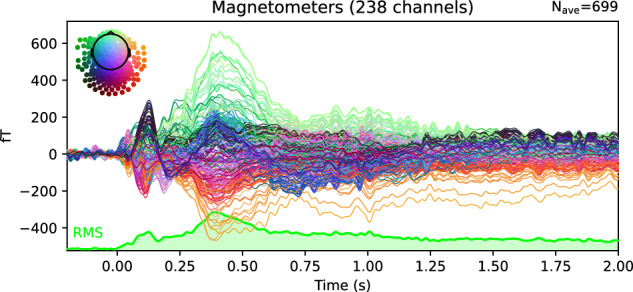
Illustration of individual Event-Related Fields per experimental condition. Each curve represents the signal amplitude (in femtoTesla, fT) of a single sensor during the trial period. The spatial arrangement of all involved sensors is shown relative to a top-view representation of the head (at the top left of the figure). The Root Mean Square (RMS) of the signals is displayed at the bottom of the figure.

**Fig. 11:**
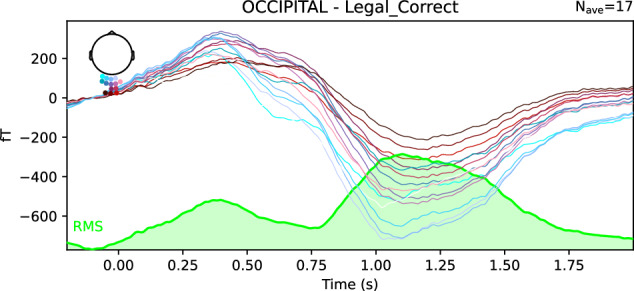
Illustration of Event-Related Fields across all subjects for one experimental condition (Legal_Correct) and one Region of Interest (occipital ROI). Each curve represents the signal amplitude (in femtoTesla, fT) of a single sensor located within the selected ROI during the trial period. The spatial arrangement of all involved sensors is shown relative to a top-view representation of the head (at the top left of the figure). The Root Mean Square (RMS) of the signals is displayed at the bottom of the figure.

**Fig. 12:**
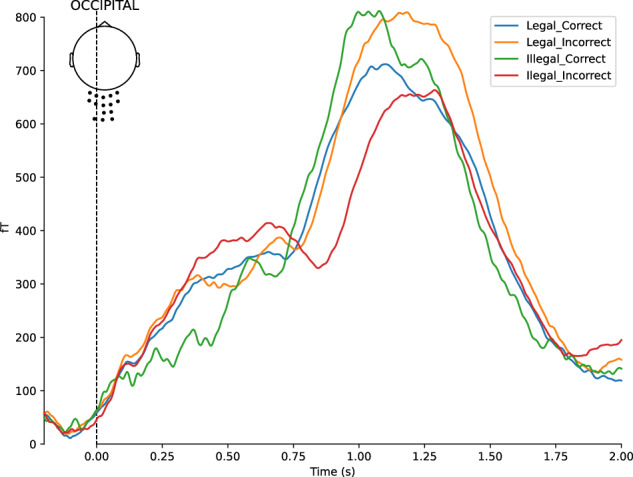
All-Subjects Root Mean Square Representation of the four experimental conditions (Legal_Correct, Legal_Incorrect, Illegal_Correct, and Illegal_Incorrect) for the occipital ROI. Each curve represents the RMS signal (in femtoTesla, fT) across sensors within the selected ROI during the trial period. The spatial arrangement of all sensors within the selected ROI is shown relative to a top-view representation of the head (at the top left of the figure).

Finally, a Grand Averaging was performed, meaning an averaging across all participants. Several figures have been generated to assess the quality of the data across all subjects:

- Whole-brain ERF representations per experimental condition, similar to the individual ERF representations shown in Fig. 10.
- ERF representations by Region of Interest (ROI) per experimental condition (Fig. 11), with three predefined ROIs, each containing approximately fifteen sensors: two temporal (left and right) and one occipital.
- Root Mean Square (RMS) representation of the four experimental conditions, either for the whole brain or by ROI (Fig. 12).

The code is shared to allow easy modification of the ROIs as needed.

- Code: /code/10_MEG_GLOUPS_GrandAveraging.py
- Input files: Epoched MEG FIF file in the participant’s /derivatives/analysis_preliminary/meg directory (e.g., sub-01_task-gloups-ave.fif)
- Output files: SVG Figures in the /derivatives/analysis_preliminary/ERFs directory

## Supplementary information

Coregistration of Anatomical (defaced MRI) and Functional (MEG) Data

## Data Availability

In the ‘code’ directory, we have included the task-specific (gloups vs rest) scripts used to generate the BIDS structure of the MEG_GLOUPS dataset. All code will be made available upon acceptance of the article for publication.
