# Supplementary material for: Dataset for Evaluating the Production of Phonotactically Legal and Illegal Pseudowords
Source: Sci Data. 2025 May 14;12:792. doi: 10.1038/s41597-025-05127-0 (PMC12078460; doi:10.1038/s41597-025-05127-0)
Supplement: Supplementary file 1 — Coregistration of Anatomical (defaced MRI) and Functional (MEG) Data [file 41597_2025_5127_MOESM1_ESM.docx]

Supplementary Data: Coregistration of Anatomical (defaced MRI) and Functional (MEG) Data

This section provides a step-by-step guide for coregistering anatomical MRI (defaced T1-weighted) and MEG data using FreeSurfer and MNE-Python. The process involves anatomical preprocessing with FreeSurfer, fiducial extraction, and alignment of MEG and MRI coordinate systems.

## Load T1w from OpenNeuro Gloups

The anatomical (defaced) T1w image can be downloaded from the OpenNeuro Gloups dataset. Ensure you have the corresponding MRI identity number (see Table 1, e.g. ID-MRI sub-04 for ID-MEG sub-01) to access to the dataset and download the required filesAfter downloading, modify the ID for consistency and verify the file integrity:

mri_info sub-01_T1w.nii.gz"

### Processing the T1w with FreeSurfer

## Before coregistration, the T1w MRI image must be processed using FreeSurfer to generate a cortical surface model and ensure proper anatomical landmarks.To preprocess the MRI data, run:

recon-all -s sub-01 -i /path/to/T1w.nii.gz -all

### Verify FreeSurfer Processing

After Freesurfer processing, check the anatomical segmentation using freeview:

freeview -v $SUBJECTS_DIR/sub-01/mri/T1.mgz \

-v $SUBJECTS_DIR/sub-01/mri/wm.mgz \

-v $SUBJECTS_DIR/sub-01/mri/brainmask.mgz \

-f $SUBJECTS_DIR/sub-01/surf/lh.white:edgecolor=blue \

$SUBJECTS_DIR/sub-01/surf/lh.pial:edgecolor=red

Make sure the surfaces correctly are aligned with the cortical structure.


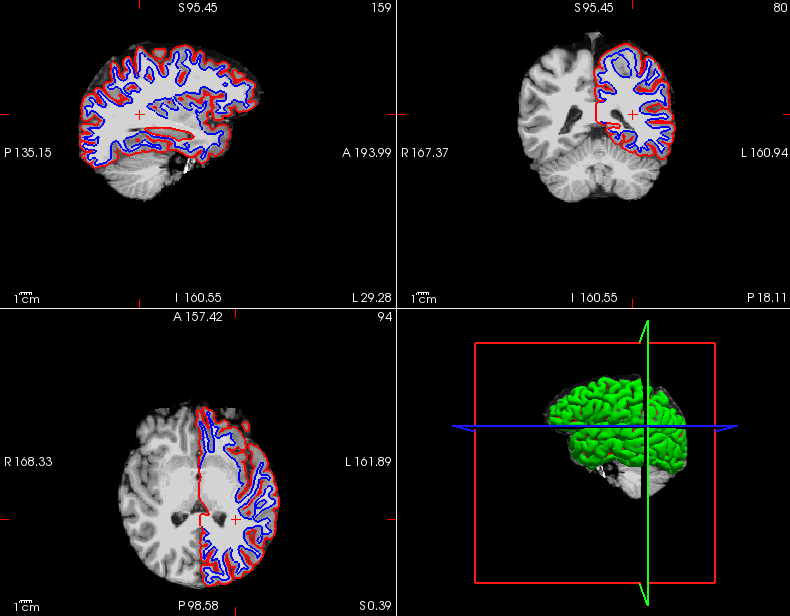


Fig.S1. Overlapping of surfaces and MRI volume in freeview

## Creating BEM Surfaces with Watershed

The Watershed algorithm provides an automated and efficient method for extracting skull and scalp boundaries, essential for forward modeling in MEG analysis. Use the following command:

mne watershed_bem -s sub-01

This command generates the necessary BEM surfaces within the subject’s Freesurfer directory (bem folder). The output includes inner_skull.surf, outer_skull.surf and outer_skin.surf).

## Coregistering MEG and MRI Data

Use MNE’s interactive coregistration tool:

mne coreg

- Load the subject’s MRI and MEG data.
- Align fiducial points (nasion, left/right preauricular points).
- Fit the head shape to the digitized scalp points.
- Save the transformation file (-trans.fif).


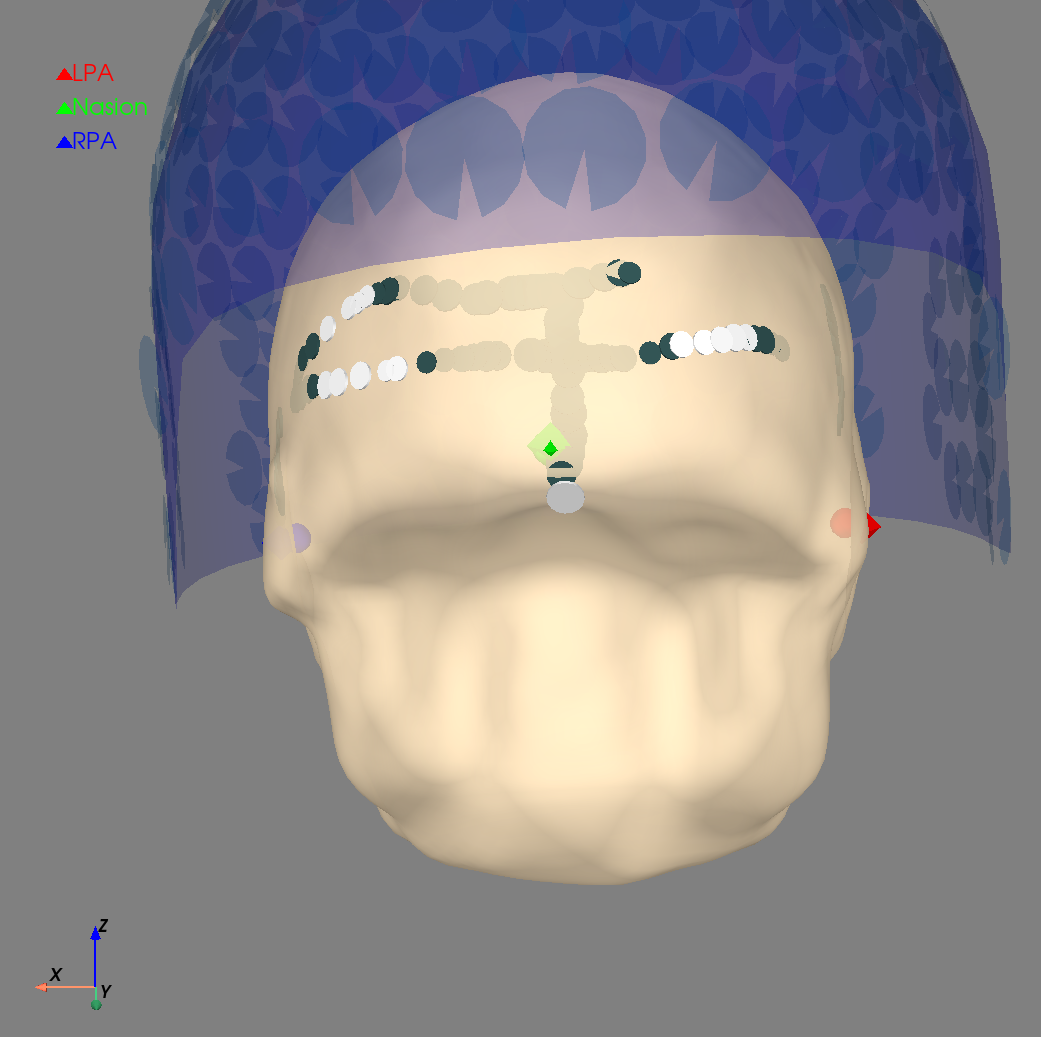


Fig.S2. A screen shot of the MNE’s interactive coregistration tool
